# Novel Balance Mechanism Participates in Stem Cell Therapy to Alleviate Neuropathology and Cognitive Impairment in Animal Models with Alzheimer’s Disease

**DOI:** 10.3390/cells10102757

**Published:** 2021-10-15

**Authors:** Chuan Qin, Yongning Li, Kewei Wang

**Affiliations:** 1 Institute of Laboratory Animal Sciences, Chinese Academy of Medical Sciences and Comparative Medical Center, Peking Union Medical College, Beijing Engineering Research Center for Experimental Animal Models of Human Critical Diseases, 5 Panjiayuan Nanli St., Beijing 100021, China; qinchuan@pumc.edu.cn; 2 Departments of International Medical Service and of Neurosurgery, Peking Union Medical College Hospital, Chinese Academy of Medical Sciences, Peking Union Medical College, Shuaifuyuan 1, Dong Cheng District, Beijing 100730, China; liyongning@pumch.cn

**Keywords:** Alzheimer’s disease, stem cell therapy, neurogenesis, synaptogenesis, astrocyte, microglia, autophagy, apoptosis, immunoregulation, neuroinflammation

## Abstract

Stem cell therapy improves memory loss and cognitive deficits in animal models with Alzheimer’s disease. The underlying mechanism remains to be determined, but it may involve the interaction of stem cells with hippocampal cells. The transplantation of stem cells alters the pathological state and establishes a novel balance based on multiple signaling pathways. The new balance mechanism is regulated by various autocrine and paracrine cytokines, including signal molecules that target (a) cell growth and death. Stem cell treatment stimulates neurogenesis and inhibits apoptosis, which is regulated by the crosstalk between apoptosis and autophagy—(b) Aβ and tau pathology. Aberrant Aβ plaques and neurofibrillary tau tangles are mitigated subsequent to stem cell intervention—(c) inflammation. Neuroinflammation in the lesion is relieved, which may be related to the microglial M1/M2 polarization—(d) immunoregulation. The transplanted stem cells modulate immune cells and shape the pathophysiological roles of immune-related genes such as TREM2, CR1, and CD33—(e) synaptogenesis. The functional reconstruction of synaptic connections can be promoted by stem cell therapy through multi-level signaling, such as autophagy, microglial activity, and remyelination. The regulation of new balance mechanism provides perspective and challenge for the treatment of Alzheimer’s disease.

## 1. Alzheimer’s Disease and Stem Cell Therapy

Alzheimer’s disease (AD) is a neurodegenerative disorder, characterized by memory decline and cognitive impairment. In pathology, AD is manifested with Aβ peptide plaques, neurofibrillary tau tangles, neuronal death, synaptic alterations, and cerebral atrophy [1,2]. The etiology of AD is complicated by the diversity of risk factors, such as heredity, aging, infection, immunity, medicines, environmental pollutants, and sociopsychological factors [3,4,5]. Certain diseases have been considered as predisposing factors for AD, such as hypothyroidism, immune-related disease, virus infection, epilepsy, depression, and schizophrenia. The early onset AD locus is located on chromosomes 21, 14, and 1, while the late-onset AD locus is on chromosome 19 [6]. The expression of typical genes such as APP, S182, STM-2 and APOE is linked with the pathogenesis of AD [7,8]. Most sporadic AD may be the result of the interaction between genetic susceptibility and environmental factors. The development of AD is associated with the comprehensive effects of various mechanisms such as oxidative stress, apoptosis, autophagy, immunity, inflammation, cholesterol metabolism, and angiogenesis [9]. Aberrant Aβ deposits and neurofibrillary tau aggregates induce neuronal death and synaptic loss. Some genes such as APOE4, ABCA7 and SLC24A4 are related to cholesterol metabolism that is implicated with P-tau trafficking [10,11]. Hence, statin drugs can decrease neurofibrillary tangle burden by competitively inhibiting HMG-CoA reductase [12]. The disturbance of neurotransmitters is connected with the clinical manifestations of AD, including acetylcholine system, monoamine system, and neuropeptides [13,14]. Immunoregulation plays an important role in the neuronal loss of patients with AD. The allelic variants of microglial TREM2 cannot control the balance between the formation and phagocytosis of Aβ proteins in the brain, increasing the risk of AD by nearly three times [15,16]. Free radical generation and oxidative stress cause neuronal apoptosis, which are related to the onset of AD as well [17].

**Figure 1:**
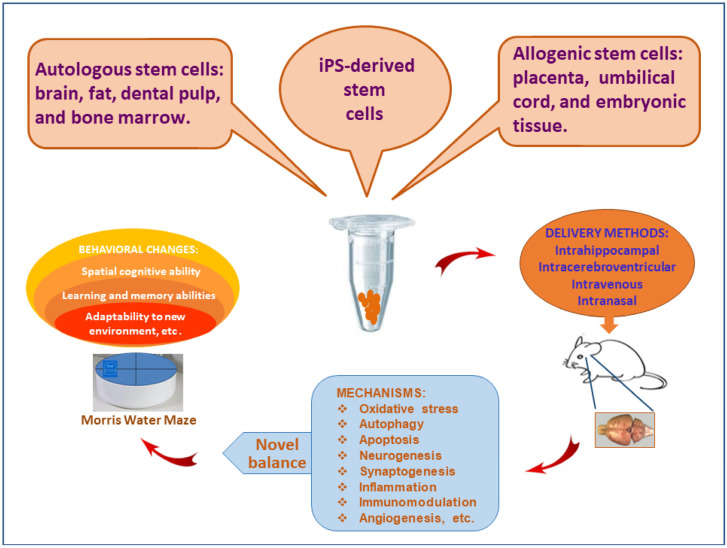
Stem cell therapy for animal models with Alzheimer’s disease. The transplantation of stem cells can stimulate the secretion of autocrine and paracrine cytokinesis, which alters microenvironment and promotes neurogenesis as well as synaptogenesis. As a result, stem cell therapy alleviates neuropathology and improves behavioral performance in animal models with Alzheimer’s disease.

The medical treatment of AD remains a challenge. Presently, only a few medicines have certain effects, including (a) acetylcholinesterase inhibitors such as donepezil, galantamine, rivastigmine, and tacrine [18]. They can compensate for the cholinergic decline by inhibiting acetylcholine turnover, (b) NMDA receptor antagonist memantine, and (c) Aβ-directed monoclonal antibody aducanumab. They target Aβ peptides to reduce their accumulation in the brain [19]. Other compounds that can reduce amyloid plaques, neurofibrillary tangles and neuroinflammation have been evaluated in clinical trials as well [20,21]. So far, no medications have been demonstrated surely to stop or delay the progression of AD. Stem cell therapy as a novel strategy has also been explored in animal models with AD (Figure 1). Research results prove that the transplantation of stem cells can improve memory and learning abilities, which can function in the AD-like animal models as reflected by extended effectiveness or longer life expectancy [9,22]. Despite the encouraging progress, therapeutic effect is expected to continue for the remaining life. Therefore, significant improvements are needed to enhance efficiency. The transplanted stem cells can proliferate and transdifferentiate, which compensate for neuronal loss and restore synaptic connection. The therapeutic mechanisms are essentially associated with neurogenesis and synaptogenesis. The source of stem cells may be autologous, allogenic, or iPS-derived [9,23,24,25,26]. Autologous stem cells can be isolated and purified from brain, fat, dental pulp, or bone marrow. In contrast, allogenic stem cells may be prepared from placenta, umbilical cord, or embryonic tissue. Additionally, the delivery methods affect the therapeutic effect of stem cells [9]. Different approaches have been compared based on feasibility and accessibility, but their therapeutic efficiency remains under investigation. It is confirmed that the transplanted stem cells can repair cognitive impairment and improve behavioral performance in AD-like animal models as demonstrated by Morris water maze test, Y-maze alternation test, plus-maze discriminative avoidance task, social recognition test, and open-field evaluation [24,27,28].

## 2. Participant Cell Types of New Balance Mechanism

**Figure 2:**
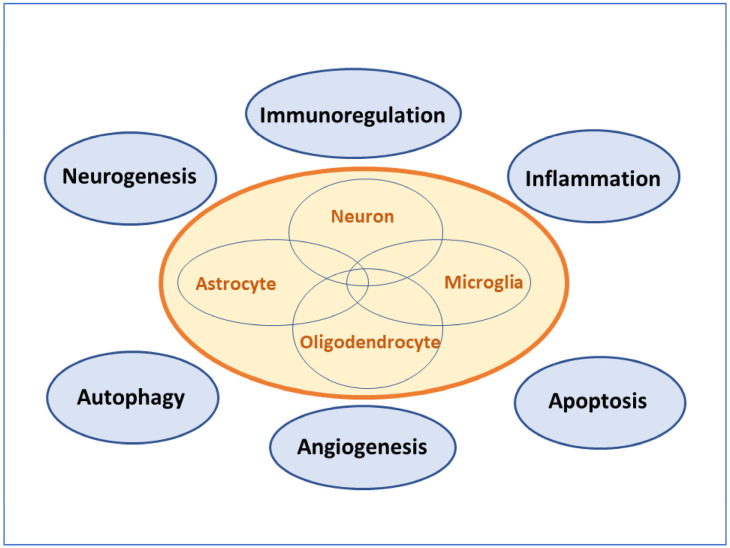
Participant cell types of a new balance mechanism. The transplantation of stem cells alters the pathological state and establishes a novel balance in the brain, which involve multiple signaling pathways such as neurogenesis, autophagy, apoptosis, inflammation, immunoregulation, the removal of aberrant proteins, neuroglial interaction, and angiogenesis. All cell types in the hippocampus participate in the establishment of the new balance mechanism. The therapeutic benefit of stem cells depends on the comprehensive effect of multi-level signaling crosstalk.

In the human brain, there are approximately 86 billion neurons and about the same number of non-neuronal glia cells [29]. The ratios of neurons to glia vary from one region to another. The transplantation of stem cells affects a variety of cell types such as neurons, oligodendrocytes, astrocytes, and microglia in the hippocampus. Intercellular interactions are regulated by different signal pathways, which bring about a series of pathophysiological changes and develop a novel balance.

(1). Physical pressure. The local pressure of cerebral tissue can be increased after the stem cells are delivered through the intrahippocampal injection, but the same phenomenon is not found via the peripheral delivery. Nonetheless, the local physical pressure caused by mechanical force is almost negligible, since a similar therapeutic effects can be obtained through tail vein delivery as well [9,30,31].
(2). Signaling molecules. The transportation of stem cells alters the microenvironment of cerebral tissue and stimulates the secretion of autocrine and paracrine cytokines, such as chemokines, leucocyte chemoattractant factors, transcription factors, inflammatory cytokines, fibrogenic cytokines, and growth factors (Table 1). Some factors are general products that can be secreted by all types of stem cells, whereas other cytokines are only produced by specific stem cells [32]. Those pragmatic cytokines participate in the establishment of new balance mechanisms. The secretion of autocrine and paracrine cytokines plays important roles in neurogenesis and synaptogenesis.
(3). Changes in various cell types (Figure 2, Table 2).
  (a) Functional neurons play a central role in the brain. There are roughly 20 billion neurons in the human cortex. Each neuron has an average 7000 synaptic connections [33]. The number of synapses is relatively stabilized in adulthood. Neuronal synapses may decrease with aging, but they can also increase due to brain plasticity. The transplanted stem cells can stimulate neurogenesis and synapse formation. Newborn neurons may be from (i) the transdifferentiation of stem cells; and (ii) the activation of specialized multipotent stem cells in the brain. At present, stem cell therapy has overcome the concerns of uncertainty and safety, and its effectiveness has been validated as well.
  (b) Oligodendrocyte is a specific subtype of neuroglia. In the central nervous system, their branch structures wrap around the neuronal axons to form an insulating myelin sheath. The physiological function of oligodendrocytes is to maintain neuronal insulation during the excitement of nerve signals. The complete structure of the myelin sheath provides a safety measure for signal transmission among neuronal synapses. Stem cell therapy restores neuronal networks by way of synaptogenesis that is protected by the myelin sheaths from oligodendrocytes [34,35].
  (c) Astrocytes, also called astroglia, have projections covering local neurons. Astrocytes are the support system in the cerebral tissue to hold neurons in the position. Additionally, they can produce cytokines and interact with other cell types. For example, astrocytes participate in microglia-mediated inflammatory and immune processes [36]. Astrocytes are responsible for substance exchange. In the CNS, astrocytes contact both capillaries and neurons to transport nutrients. Moreover, the phagocytosis of astrocytes is implicated in the amyloid load of Alzheimer disease [37]. In the process of stem cell therapy, the precise roles of astrocytes are still unclear. After exposure to MSC-conditioned medium, the expression of pro-inflammatory factors such as IL-1β, TNF-α and IL-6 was attenuated in cultured astrocytes [38]. The transplanted stem cells acted on astrocytes to modify neuroimmune and relieve neuroinflammation in vivo [39].
  (d) Microglia are resident immune cells in the brain, equivalent to macrophages. Functional microglia take part in the neuroinflammation, immunomodulation, the elimination of Aβ proteins, and tau pathology. As the first line of the neuroimmune system, microglia remove cerebral debris and protect neurons from harmful invasion. In contrast, the inflammatory factors released by microglia can cause receptor-induced neuronal apoptosis [40]. Fortunately, microglial activity can be modulated by the transplanted stem cells. So, stem cell therapy suppresses neuroinflammation and controls neuroimmune overreaction. Furthermore, microglia can detect neuronal injury and play a critical role in the maintenance of neuronal health. As immune cells, microglia have duality in the pathogenesis of AD. They can not only protect neurons by engulfing detrimental Aβ proteins, but also damage neurons by secreting inflammatory cytokines [41,42,43]. The consequence may be beneficial or pernicious, which is determined by the comprehensive effect of multi-level signaling crosstalk.

## 3. Representative Signaling Pathways of New Balance Mechanism

The transplantation of stem cells alters the pathological state and stimulates the secretion of autocrine and paracrine cytokines in the hippocampus. The remodeling process establishes a novel balance related to multiple signaling pathways. The new balance is an essential mechanism to improve the neuropathology and recognitive deficits of Alzheimer’s disease, which has been validated by regulating representative pathways.

### 3.1. The Transplantation of Stem Cells Mediates Cell Growth and Death

**Figure 3:**
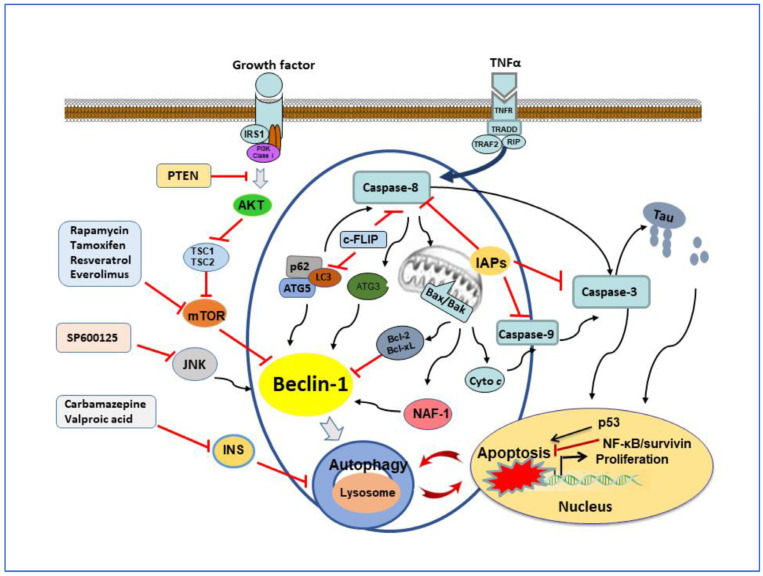
Crosstalk between autophagy and apoptosis. Cell fate is regulated by the interaction between autophagy and apoptosis. There is a crosstalk between apoptosis and autophagy by sharing common regulators, such as p53, Atg5, caspase-8, Beclin-1/Bcl-2, and IAPs. Cellular FLIP inhibits caspase 8 and autophagosome formation that is mediated by LC3 conjugation. Autophagosome promotes the activation of caspase 8 through the platform consisting of ATG5, LC3 and p62. Bcl-2 family involves both autophagy and apoptosis by regulating signal molecules such as Beclin1 and BAX/BAK dimer. The activation of autophagy can degrade IAPs to facilitate apoptosis. Activated caspase-3 causes apoptosis but suppresses autophagy. The red line represents the inhibitory effect.

The transplanted stem cells can survive in the hippocampus and further transdifferentiate into neurons as demonstrated in APP/PS1 transgenic mice [24,26]. Meanwhile, some newborn neurons may be derived from endogenous progenitors, which have been detected in C57BL/6 mice as well as in the tissue culture of a patient’s cortex [44,45]. More details of in vivo conditions still need to be verified on the patient. Further, the beneficial cytokines produced by MSCs can stimulate proliferation through the indirect regulation of neurotrophic factors such as NGF, FGF2 and BDNF [46]. The comprehensive effect of transplanted stem cells is to promote neuronal growth or neurogenesis. Generally, the development of DA is presented with long-term and gradual characteristics, accompanying neuronal apoptosis/necroptosis/necrosis. Apoptosis is an important way of neuronal death, especially at the early stage of AD. Apoptosis is initiated in a controlled environment. Apoptotic body may be promptly removed via phagocytosis. Thereupon, histopathological changes are slight or insignificant. Tissue biopsy may be the only way to confirm the apoptosis in most cases. Perhaps, this is the reason that the low rate of apoptosis is observed in some stages. Apoptotic cell death rarely exhibits acute features such as inflammatory necrosis caused by microbial infection or thrombosis. The transplanted stem cells stimulate neurogenesis and inhibit apoptosis-related neuron death [9,47]. Besides, stem cell therapy decreases the generation of ROS and alleviates ROS-induced neuronal damage. Interestingly, short-term ROS exposure promotes the proliferation of neural progenitors whereas persistent ROS stimulation aggravates oxidative stress and neuronal apoptosis [48]. The transplanted stem cells may control the dynamic equilibrium between ROS generation and elimination, thereby regulating neurogenesis. In clinical, the oxidative damage in the advanced AD is very severe, leading to neuronal loss and cognitive decline [49]. The transplanted stem cells activate autophagy in AD-like animal models. The activation of autophagy is reflected by the upregulation of BECN1/Beclin 1 and the increased number of LC3-II-positive autophagosomes in the hippocampus, which boosts the clearance of Aβ peptides and the relief of oxidative stress [17]. Autophagy is a key mechanism to promote neurogenesis as demonstrated by the expression levels of signal molecules such as Beclin-1, atg5, LC3-II, and mTOR. The proliferation of neural progenitor cells in adult hippocampus is regulated by the PI3K/AKT/mTOR and ERK1/2 signaling pathways [50,51,52]. There is a crosstalk between autophagy and apoptosis (Figure 3). The induction of autophagy is begun while Beclin-1 is dissociated at the BH3-only domain of Bcl-2 proteins subsequent to the phosphorylation of Bcl-2. Activated autophagy alleviates neuronal apoptosis by altering the levels of IAPs, Bcl-2, caspase-8 and so forth. The autophagic response can be balanced by caspase activation. Activated caspase-8 cleaves Beclin-1 into C-terminal and N-terminal fragments to trigger apoptosis [53]. The cell fate is modified by the interaction of diverse BH3 proteins with Beclin-1 and caspase-8 [54]. The beneficial effect of transplanted stem cells may be through the upregulation of BECN1/Beclin-1, the modulation of Bcl-2 family, and the inhibition of caspase activity [22,55]. The crosstalk between autophagy and apoptosis modulates the therapeutic effect of transplanted stem cells. A synergistic effect may be acquired when the transplanted stem cells is combined with autophagic and/or apoptotic mediators.

### 3.2. The Transplanted Stem Cells Regulate the Production and Removal of Aberrant Proteins

There are aberrant Aβ proteins and tau aggregates in the brain. Both Aβ plaques and tau tangles are increased with advanced age and/or genetic factors. The buildup of two proteins is associated with the pathogenesis of Alzheimer’s disease, although the causal connection remains to be determined. Owing to the hindrance of Aβ metabolic pathway with aging, the production of Aβ proteins, especially insoluble Aβ proteins, is more than their degradation. The Aβ peptides form plaques to deposit in the brain of patients with AD. Genetic modification demonstrated that the down-regulation of Becn-1 increased extracellular Aβ deposition, whereas the high expression of Beclin-1 decreased the Aβ pathology in APP transgenic mice [54,56,57]. The reversal relationship provides the mechanistic link between autophagic Beclin-1 expression and cytotoxic Aβ deposits. Aβ proteins is derived from γ-secretase-hydrolyzed APP [58,59]. Simultaneously, γ-secretase also activates Notch receptors for Aβ metabolism [60,61]. Aging weakens the activation of the Notch signaling pathway and leads to the accumulation of hydrolyzed APP, which is closely related to the pathogenesis of AD. In addition, there is evidence that aberrant Aβ proteins can inhibit the PI3K/Akt signaling pathway and autophagic activity [62,63]. Cytotoxic Aβ proteins can induce the apoptosis of primary cultured neurons. Furthermore, the injection of Aβ proteins into the hippocampus produces AD-like manifestations in animal models, showing similar changes to AD patients [64]. The accumulated Aβ proteins launch apoptotic, necroptotic, and necrotic mechanisms. Aβ-mediated cytotoxicity causes irreversible damage during cell maturation, which impairs neurogenesis by decreasing the survival rate of newborn neurons [65,66]. As a consequence, the integration of newly generated neurons into the hippocampal circuitry is decreased, resulting in the decline in learning and memory capabilities. Immunotherapy with antibodies targeting Aβ proteins have been explored in clinical trials [67]. Obviously, aberrant Aβ deposits and weak neurogenesis are related to the pathogenesis of AD. Aging as a risk factor complicates the metabolism of AD-associated Aβ proteins [66]. Meta-analysis revealed that the transplantation of stem cells could decrease Aβ plaques in the hippocampus of APP/PS1 mice, which promoted the functional improvement of AD-like animals [9]. Sometimes, stem cell therapy cannot significantly decrease Aβ plaques in certain studies. Furthermore, certain drugs diminish Aβ protein load but may not ameliorate memory loss and cognitive deficits. Thus, the theory of Aβ pathology is controversial. Neurofibrillary aggregates are formed by the hyperphosphorylation of microtubule-associated protein tau. Tau tangles are composed of tubular filaments, paired helical filaments, and hyperphosphorylated tau protein, which are associated with the decreased autophagy [68,69,70]. The intracellular accumulation of tau tangles can cause ER stress-induced apoptosis, but tau hyperphosphorylation may also induce apoptotic escape and initiates neurodegeneration [48,68,71]. The expression of JNK in the hippocampus and cortex of AD patients was exceedingly increased [72,73]. In rapidly aging mice with AD, the JNK cascade was dramatically higher than that in normal mice [74]. JNK may involve the regulation of tau protein via oxidative stress. The inhibition of JNK phosphorylation can decrease the level of phospho-tau proteins. AD-like tau pathology and cognitive impairment are exacerbated by reducing insulin/GSK-3β signaling activity [75]. Tau hyperphosphorylation and the CaM-CaMKIV signal pathway participate in the recovery of memory ability in AD-like rats [76]. The transplantation of stem cells can lower tau aggregates and inhibit neuronal apoptosis. Moreover, reduced tau tangles are beneficial to both young and aged AD-like animals [9,77,78]. The improvements of the aforementioned neuropathology are related to the enhancement of autophagy [79,80]. Clearly, stem cell therapy not only facilitates the elimination of aberrant proteins, but also prevents their formation. These are two different aspects that transplanted stem cells can deal with.

### 3.3. The Transplanted Stem Cells Can Produce Pro- and Anti-Inflammatory Cytokines

**Figure 4:**
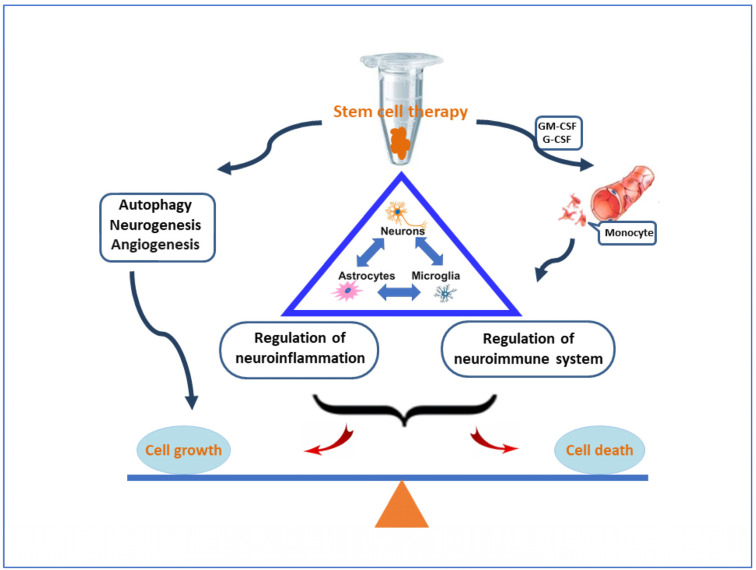
Regulation of inflammatory and neuroimmune responses. The transplanted stem cells inhibit neuroinflammation and participate in immunoregulation. Moreover, peripheral monocytes can be recruited to accelerate the removal of aberrant proteins. The signaling pathways of new balance mechanism form a complex network, but inflammatory/immune processes are key regulators to determine neurogenesis and synaptogenesis, which play a critical role in the pathogenesis of Alzheimer’s disease.

Inflammation is a response to a variety of stimuli such as infection, toxic metabolites, and autoimmunity. The initiation of neuroinflammation may be a protective action, but the actual consequence leads to harmful tissue damage. The triggers of neuroinflammation can be cytokines, metabolites, or aberrant Aβ proteins. A lot of evidence supports that neuroinflammation is an independent factor affecting the different stages of AD. Inflammatory cytokines, small molecular proteins secreted by glial cells in the brain, are key factors by binding to corresponding receptors on the cell surface. It was found that thirteen pro-inflammatory cytokines in patients with AD, including IL-1β, IL-6, IL-18, TNF-α and so on, were significantly higher than those in the normal control [81,82]. Conversely, some anti-inflammatory cytokines play a protective effect in the pathogenesis of AD. For instance, IL-10 is the primary product of active monocytes. Its functions include phagocytosis, the expression of Th1 cytokines, the regulation of costimulatory molecules, and MHC class II antigen presentation [83]. IL-10 can inhibit inflammation by blocking the cytotoxicity of pro-inflammatory cytokines. The IL-10/STAT3 signal pathway can be regulated to rebalance the natural immunity in the brain, which may bring about beneficial effects on neuroinflammation [84]. The signal components in the classic IL-10 pathway are up-regulated in the hippocampus of AD patients. Besides, the cytokines IL-2 and IL-4 have anti-inflammatory effects similar to IL-10 [40,85]. Distinctly, inflammatory cytokines have protective and harmful effects. The NF-κB signal pathway is related to inflammation, oxidative stress, and apoptosis in the brain [86]. The cerebral levels of BACE1 and NF-κB p65 are markedly enhanced in patients with AD. Anti-inflammatory drugs or stem cell therapy can block the transcription of BACE1 as well as the production of Aβ, suggesting that the inhibition of NF-κB-mediated BACE1 expression is the plausible target of AD treatment [87]. There are complicated interactions among hippocampal cells such as astrocytes, neurons, and microglia (Figure 4). Neurons are functional carriers in the brain, implicated in the inflammatory response by producing Aβ deposits and tau tangles [88]. Meanwhile, neurons are also targets that need to be protected during neuroinflammation. Astrocytes provide support, protection, and nutrient supply to neurons under physiological conditions. Active astrocytes can secrete inflammatory cytokines, such as RANTES, MIP-1α, MCP-1 and complement, to participate in the neuroinflammation [36,89]. The transplanted stem cells may suppress inflammation caused by astrocytes [90]. Pro-inflammatory factors such as IL-1β, TNF-α, and IL-6 was decreased in cultured astrocytes following exposure to MSC-conditioned medium [38]. Microglia are the innate immune cells of the central nervous system. There are fine-tuning mechanisms for microglia to protect cerebral neurons. They can remove aberrant Aβ protein plaques. Additionally, microglia are able to maintain neuronal connections as well as modulate the electrical activity. Microglial activity regulates neuronal function and vice versa. However, the above-mentioned relationship is interrupted in the pathogenesis of AD. The dysfunction of neuronal conduction is a prominent feature, leading to cognitive deficits. The pathogenic role of microglia in development of AD is demonstrated by genetic mutations [91]. The abnormal interaction between neuronal and microglial activities is engaged in the active cycle that deteriorates cognitive impairment. Microglial activation has a duality in the pathogenesis of AD. They protect neurons by engulfing detrimental substances and attack neurons by secreting inflammatory cytokines. The dual role of microglia may be due to the polarization of M1/M2 phenotype [92,93]. The classic M1 state can be activated by Aβ deposits to produce pro-inflammatory factors such as TNF-α, IL-1β, IFN-γ, thereby exacerbating inflammatory cell death [42,43]. The M2 phenotype may generate anti-inflammatory factors such as IL-2, IL-4 or IL-10, facilitating cell repair and neuroprotection [94,95,96]. In the APP/PS1 transgenic models, the profiles of gene expression are overlapped between microglial M1 and M2 types. Accordingly, the exact role of microglia has not yet been determined. Both the beneficial and detrimental effects of microglia can be fulfilled in the pathogenesis of AD. Available data demonstrate that the transplanted stem cells take part in the regulation of immune and inflammatory processes. After the administration of stem cells, microglial activation stimulates the removal of Aβ deposits and neuroinflammation is thereupon alleviated. Consequently, stem cell therapy can suppress inflammation. Furthermore, stem cells can recruit peripheral monocytes across blood–brain barrier into the lesion. The activation of the newly recruited monocytes can further accelerate the clearance of Aβ peptides as well as apoptotic bodies. This phenomenon seems contradictory, but it does happen. Still, many intermediate details need to be clarified through future research. Nevertheless, the comprehensive effect of stem cell therapy is conducive to the improvement of neuropathology as well as cognitive impairment in Alzheimer’s disease.

### 3.4. Immunoregulation Is Modulated by the Transplanted Stem Cells

The CNS is immunologically privileged, since peripheral immune cells are usually blocked by the blood–brain barrier composed of astrocytes and endothelial cells. Pathological studies have revealed that viral, bacterial, and fungal infections are related to the pathogenesis of Alzheimer’s disease. For example, HSV-1 DNA was found within amyloid plaques [97]. *Borrelia burgdorferi* bacterium caused Lyme neuroborreliosis and dementia [98]. The diffuse mycosis was related to the development of Alzheimer’s disease. Further studies proved that fungal infections could occur in different brain regions of patients with AD, but are absent in the control individuals [99]. The pathogenesis of Alzheimer’s disease may be partly explained by the microbial infection of CNS due to immunodeficiency, but this pathogen hypothesis needs more evidence to confirm the causality. It is well known that APOE4 and TREM2 variants associated with the development of AD may be susceptible to HSV-1 infection [100,101]. Another possibility is that both gene variants and HSV-1 infection are related to the pathogenesis of AD. In addition, the immune system decreases its protective capacity with aging. Advanced age (e.g., over 59 years old) significantly increased the mortality in patients with Alzheimer’s disease after SARS-CoV-2 infection [102,103]. Therefore, aging is a predominant risk factor related to AD [104]. The dysfunction of the immune system in the brain is demonstrated by the partial mutations of TREM2 and CD33 genes [15,105]. In patients with AD, aberrant Aβ proteins activate T cells, perpetuating the cycle of immune-mediated cell injury and repair [106]. The neuroimmune and immunoregulation are the basic targets of understanding the pathogenesis of AD. The activation of microglia participates in immunoregulation in the pathogenesis of AD. As innate immune cells in the brain, microglia have functions similar to macrophages and can be activated in response to microbial infections and toxic metabolites. Their effects on immunoregulation had been verified using the CRISPR knockout method [107]. Microglia protect the brain and maintain neuronal health by removing aberrant Aβ plaques as well as apoptotic bodies. Microglia contain the M1/M2 phenotype, playing a dual role in the pathogenesis of AD. Therefore, only immunosuppression or immunoenhancement cannot acquire beneficial effects on the development of AD. Perhaps, the damaged neuroimmune in the AD brain needs to be rebalanced. The IL-10/JAK1/STAT3 signaling pathway can regulate the establishment of immunobalance in the brain [84,108]. When bone marrow stem cells were transplanted into immunodeficient mice with AD, the transfused stem cells could restore missing immune cells for the elimination of Aβ plaques [109,110]. The transplanted stem cells can (a) inhibit microglial activation and neuroinflammation and (b) recruit peripheral monocytes across the blood–brain barrier into the lesion. These monocytes may switch the microglial M1/M2 phenotype to accelerate the removal of Aβ plaques in the AD brain [111,112,113] and (c) secret cytokines. Certain cytokines released by MSCs can facilitate cell survival and proliferation through the regulation of NGF, FGF2 and BDNF [46]. The transplanted stem cells promote neurogenesis and inhibit neurodegenerative cell death. Of note, a variety of autocrine and paracrine factors produce distinct functions. Some cytokines take part in relevant pathways to relieve neuropathology, but other factors are competitors or bystanders. Therefore, the pathophysiological roles of autocrine and paracrine factors should be scrutinized in future studies. Moreover, the expression of immune-related genes is modulated by transplanted stem cells, including TREM2, CR1, HLA-DRB5, CD33, MS4A, INPP5D, EPHA1, and CLU (Table 3). These genes influence the different stages of AD and play a crucial role in the pathogenesis of AD. The dysfunction of immune-related genes can be corrected by stem cell therapy, which has been demonstrated in AD-like models [9,109,110]. Microarray analysis and high-throughput gene sequencing have confirmed the gene profiles. Evidently, immune factors do participate in the pathogenesis of Alzheimer’s disease. The immunoregulation can effectively alleviate neuropathology and improve cognitive function. Noticeably, the transplanted stem cells are neither immunosuppressant nor immunostimulant, but they function as a regulator or controller that balances the neuroimmune response to maintain neuronal health.

### 3.5. The Transplanted Stem Cells Participate in Synaptic Plasticity

The change of neuronal synapses is pivotal pathway to the new balance mechanism. Patients with AD show a decrease in the number of synapses. After stem cell treatment, the favorable improvement is verified by increasing the number of synapses [23,26,114]. Moreover, the synthesis of neurotransmitters is also enhanced, which is consistent with the effect of neurotransmitter drugs. The enhancement of the quantity and quality of neuron synapses may explain why stem cell therapy can improve the cognitive symptoms of AD-like animal models. The formation of synapses (synaptogenesis) in the nervous system covers the lifespan of healthy individual. This process is an essential requirement for maintaining the normal function of nerve activity. There is a certain degree of synaptic pruning between neurons and synapses through competition for neural growth factors. Therefore, synaptogenesis is regulated by autocrine and paracrine cytokines. The secretion of cytokines establishes a precise relationship between synaptogenesis and microglial activity. Microglia play an important role in protecting neuronal connections and maintaining the integrity of neural circuits. Microglia have a direct role in modulating the electrical activity of neurons. The presence of aberrant proteins and/or toxic factors can damage microglial function. The protective effect of microglia may thus be impaired. At this moment, dysfunctional microglia can hurt synaptic connections. The dysfunction of synaptic networks incur cognitive deficits in Alzheimer’s disease. Canonical Wnt signal transduction involves the early neurodevelopment in the brain and the maturation of the blood–brain barrier. Wnt/β-catenin signaling regulates synaptic plasticity and the development of acetylcholine receptors, which may be related to the pathophysiology of AD [115,116]. Meanwhile, Aβ proteins can activate GSK3, thereby promoting the phosphorylation of tau protein as well as reducing the activity of Wnt [117,118,119]. Previous studies demonstrate that WASP-1 may significantly improve memory and synaptic transmission. The transplantation of stem cells can decrease aberrant Aβ peptides and tau aggregates to facilitate synaptogenesis. As proved in the iPS cells of AD patient, synaptogenesis is associated with lysosomal vacuolar-type H-ATPase and intracellular Ca^2+^ concentration. The impairment of autophagy inhibits synaptogenesis and neurogenesis [120]. The transplantation of neural stem cells stimulates cellular changes and improves behavioral performance, which may be attributed to the recovery of synaptic connectivity through neurotrophin release (i.e., GAP-43, BDNF) [121]. In addition, endogenous neural regeneration is enhanced by mobilizing the NCAM-derived peptide FG loop to amplify remyelination as well as modulate neuroinflammation [122,123].

## 4. Perspective

### 4.1. Therapeutic Efficiency and Synergistic Effect

The synergistic effect between neurotrophic cytokines and stem cells may increase therapeutic efficiency. The transplantation of stem cells can enhance neurotrophic factors such as BDNF and NGF [34,124,125,126]. Neurotrophic BDNF is related to the canonical nerve growth in the brain. NGF is a prototypical growth factor, involved in numerous biological processes such as the survival of target neurons, and the regulation of proliferation and neuroimmune. Therefore, the application of therapeutic stem cells may be pretreated with neurotrophic factors to produce synergistic effects.

### 4.2. Stem Cell Viability

There is a low survival of transplanted stem cells in the recipients, which is a real problem in therapeutic practice. Anyway, it can be improved by activating autophagy in stem cells. Novel strategy may consider that the transplanted stem cells are combined with the nanoparticles of autophagy-enhancing agents and/or apoptosis regulators, especially for the treatment of advanced AD.

### 4.3. The Improvement of Delivery Methods

Clinically, mannitol infusion is often applied to reduce intracranial pressure. The intra-arterial infusion of mannitol can transiently open the blood–brain barrier by loosening tight junctions. This technique can be utilized for stem cell delivery. After the blood–brain barrier is opened, stem cells may be transfused through the peripheral vein instead of intracranial injection. In addition, the intranasal delivery of stem cells can acquire functional improvement in the APP/PS1 models of AD [127].

### 4.4. Exosomes

In the process of stem cell therapy, the details of exosomes produced by stem cells are unknown [128,129,130]. It is possible that the exosomes of stem cells stimulate the secretion of autocrine or paracrine cytokines to achieve therapeutic effects. Accordingly, the role of exosomes needs to be clarified through future analysis.

## 5. Challenges

(1). The selection of surveillance biomarkers. Currently, monitoring markers (i.e., Aβ42, T-tau and P-tau, or exosomes in cerebrospinal fluid and/or peripheral bloodstream) need to be optimized for the evaluation of therapeutic effects.
(2). The timeline of the new balance mechanism. Following the transplantation of stem cells, the pathological state is altered and then a new balance is developed. However, it is unsure how long the dynamic reconstruction can be maintained. Perhaps, it is necessary to repeatedly transplant stem cells to obtain reliable therapeutic effects. At this time, it is important to optimize the relevant parameters of stem cell transplantation, including cell concentration, time interval, inoculation position, and delivery method.
(3). Uncertainty and perplexity. The therapeutic effect of transplanted stem cells involves multiple mechanisms, such as immunomodulation, inflammation, apoptosis, neurogenesis, autophagy, and angiogenesis. The integration of various mechanisms establishes a new balance and brings about beneficial improvements. Nowadays, most of the above-mentioned mechanisms have been investigated and their roles have been elucidated. Nevertheless, the details of relevant mechanisms still need to be explored, such as autophagy and immunomodulation, the interaction between astrocytes and microglia, microglial activation and synaptic remodeling, etc.

In summary, stem cell therapy is beneficial to the improvement of animal models with AD, which is demonstrated by the alleviation of neuropathology and the amelioration of cognitive impairment. The transplantation of stem cells alters regional microenvironment by stimulating the secretion of autocrine and paracrine cytokines, which promotes neurogenesis as well as synaptogenesis. Potential mechanisms are associated with autophagy, apoptosis, the elimination of aberrant proteins, the interaction of different neuroglia, inflammation, and immunoregulation. Those functional activities alter the pathological state and establish a novel balance by integrating multiple signal pathways. The new balance mechanism is the comprehensive effect of multi-level signaling crosstalk in the brain, which not only lays a theoretical foundation for stem cell therapy but also provides perspectives and challenges for the treatment of Alzheimer’s disease.

## Figures and Tables

**Table 1:** Autocrine and paracrine cytokines secreted by stem cells.

Types	Examples	Function	References
**Inflammatory cytokines**	TNFα, IL-1, IL-2, IL-6, IL-10	To regulate inflammatory and immune responses, to participate in the regulation of cell growth and apoptosis, etc.	J. Clin. Endocrinol. Metab. 1998 Jun;83(6):2043–51; Immunotherapy. 2018 Sep;10(12):1053–1064;
**Fibrogenic cytokines**	FGF, TIMP-1	Proliferation of fibroblasts, collagen synthesis and extracellular fibrosis, immune mediators.	PLoS ONE. 2019 Apr 22;14(4):e0215678; Brain Res. 2004 Apr 16;1005(1–2):21–8.
**Chemokines**	CCL5, CXCL-10, CXCL-12,	Chemo-attractants, to guide the migration of cells, to regulate immunity, inflammation, angiogenesis, etc.	Stem Cells. 2012 Jul;30(7):1544–55; Cancer Res. 2011 Jun 1;71(11):3831–40; J. Cell Physiol. 2019 Aug;234(10):18707–18719
**Leucocyte chemoattractant factors**	CINC-1, G-CSF, SCF, GM-CSF	To participate in immune/inflammatory cascade.	PLoS ONE. 2019 Apr 22;14(4):e0215678; Blood. 2000 Nov 15;96(10):3422–30.
**Transcription factors**	GATA-4, Nkx2.5, MEF2C	Response to intercellular and extracellular signals, transcriptional regulation in development, cell cycle, and pathogenesis.	Mol. Med. Rep. 2015 Aug;12(2):2607–21; Tissue Eng. Part A. 2011 Jan;17(1–2):45–58.
**Growth factors**	HGF, IGF-1	Signaling molecules promote cell differentiation and maturation.	Stem Cells Dev. 2010 Jul;19(7):1035–42; Int. J. Stem Cells. 2009 May;2(1):59–68.
**Vascular endothelial growth factor**	VEGF	To stimulate the formation of blood vessels.	Int. J. Stem Cells. 2009 May;2(1):59–68; Brain Res. 2004 Apr 16;1005(1–2):21–8.
**Other**	MCP-1, OPG	Selectively recruiting monocytes, to regulate bone metabolism.	Int. J. Stem Cells. 2009 May;2(1):59–68; J. Interferon Cytokine Res. 2009 Jun;29(6):313–26; Cell. 1997 Apr 18;89(2):309–19.

**Table 2:** New balance mechanism in the hippocampus involves multiple signaling pathways.

Mechanisms	Cell Types	Signaling Pathways	References
**Immunoregulation**	Neurons, Microglia, Astrocytes, Oligodendrocytes	To facilitate microglial M1/M2 polarization; to regulate the crosstalk between T cells and microglia; to mediate synaptic plasticity.	Neuroscience. 2019 Dec 1;422:99–118; Proc. Natl. Acad. Sci. USA. 2006 Mar 28;103(13): 5048–5053; Front. Synaptic Neurosci. 2018 Jun 13;10:14.
**Inflammation**	Neurons, Microglia, Astrocytes, Oligodendrocytes	To decrease the level of NF-κB in astrocytes; to reduce the levels of TNF-α, IL-6, and MCP-1; to regulate cell growth and apoptosis.	Neuropathol. Appl. Neurobiol. 2017 Jun;43(4):299–314; Sci. Rep. 2020 Jul 1;10(1):10772; DOI:10.1186/s13024-015-0035-6.
**Neurogenesis**	Neurons, Microglia, Astrocytes, Oligodendrocytes	To increase IGF-1 expression in the hippocampus; to increase N-acetylaspartate and Glutamate; to induce the expression of synaptophysin.	Exp. Ther. Med. 2017 Nov; 14(5): 4312–4320; Transl. Neurodegener. 2020 May 27;9(1):20; Hippocampus. 2017 Dec;27(12):1250–1263
**Autophagy**	Neurons, Microglia, Astrocytes, Oligodendrocytes	To increase cellular viability and LC3-II expression; to upregulate BECN1/Beclin 1 expression; to enhance mitophagy.	Autophagy. 2014 Jan;10(1):32–44; Mol. Neurobiol. 2019 Dec;56(12):8220–8236; Autophagy. 2021 Jan 19;1–20.
**Apoptosis**	Neurons, Microglia, Astrocytes, Oligodendrocytes	To regulate expression of hippocampal SIRT1, PCNA, p53, ac-p53, p21, and p16; to target caspase pathway; Ca^2+^ signaling.	Behav. Brain Res. 2018 Feb 26;339:297–304; Front. Neurosci. 2018 May 22;12:333; Curr. Alzheimer Res. 2010 Sep;7(6):540–8; Sci. Rep. 2016 Aug 12;6:31450.
**Angiogenesis**	Neurons, Microglia, Astrocytes, Oligodendrocytes	BMSCs secrete VEGF, BDNF, NT-3, IGF-1, bFGF, GDNF and TGF. VEGF is the most important mitogen in the process of angiogenesis.	Brain Res. 2011 Jan 7; 1367:103–113; Int. J. Mol. Med. 2013 May;31(5):1087–96; Neuroreport. 2015 May 6;26(7):399–404.
**Synaptogenesis**	Neurons, Microglia, Astrocytes, Oligodendrocytes	To stimulate the production of BDNF and NGF for remyelination; peptide FG loop (FGL) amplifies remyelination and modulates neuroinflammation.	Cell Biol. Int. 2021 Feb;45(2):432–446; J. Neuroimmune. Pharmacol. 2016 Dec;11(4):708–720; Front. Cell Dev. Biol. 2021 Jul 2;9:680301.

**Table 3:** Immune-related genes are implicated in the pathogenesis of Alzheimer’s disease.

Names	Function	References
**TREM2**	Transmembrane glycoprotein. To mediate immune and inflammatory responses as microglial receptor.	Neurobiol. Dis. 2020 Nov;145:105072; Neurobiol. Dis. 2019 Jul;127:432–448.
**CR1**	To regulate complement cascade and mediate immune adherence as well as phagocytosis.	Stem Cell Res. 2016 Nov;17(3):560–563.
**HLA-DRB5**	To encode major histocompatibility complex class II protein involved in immune responses.	Neurol. Genet. 2018 Jan 18;4(1):e211; JAMA Neurol. 2015 Jan;72(1):15–24.
**CD33**	Microglial receptor converged on immune-inflammatory pathways.	Neurobiol. Dis. 2019 Jul;127:432–448; Gerontology. 2019;65(4):323–331
**MS4A**	Belonging to a class of four-transmembrane spanning proteins.	Aging Cell. 2019 Aug;18(4):e12964.
**INPP5D**	At the plasma membrane, the protein hydrolyzes the 5′ phosphate and regulates multiple signaling pathways.	EMBO Mol. Med. 2020 Mar 6;12(3):e10606.
**EPHA1**	To regulate the developmental of nervous system.	Int. J. Comput. Biol. Drug Des. 2020;13(1):58–70; J. Immunol. 2020 Sep 1;205(5):1318–1322.
**CLU**	Diverse functions such as protein chaperoning, apoptosis, complement activation, etc.	Mol Neurodegener. 2015 Jul 16;10:30; Turk J Med Sci. 2015;45(5):1082–6.

## Abbreviations

AD, Alzheimer’s disease; Aβ, amyloid beta or β-amyloid; APP, amyloid-beta precursor protein; iPS cells, induced pluripotent stem cells; BACE1, β-site APP cleaving enzyme 1; HSV-1, herpes simplex virus type 1; WASP-1, Wnt activating small protein; BDNF, brain-derived neurotrophic factor; NGF, Nerve growth factor; FGF2, fibroblast growth factor 2; TREM2, triggering receptor expressed on myeloid cells 2; CR1, complement receptor type 1; MS4A, membrane-spanning 4A; INPP5D, inositol polyphosphate-5-phosphatase D; EPHA1, ephrin type-A receptor 1; CLU, clusterin; HLA-DRB5, HLA class II histocompatibility antigen, DR beta 5; IL-1β, interleukin 1β; IL-10, interleukin-10; TNF-α, tumor necrosis factor α; IFN-γ, interferon γ; GAP-43, growth-associated protein 43; NCAM, neural cell adhesion molecule; MSC, mesenchymal stem cell; CNS, central nervous system; ROS, reactive oxygen species; Bcl-2, B-cell lymphoma 2; LC3, microtubule-associated proteins 1A/1B light chain 3B; LC3-II, lipid modified form of LC3; IGF-1, insulin-like growth factor 1; IAPs, inhibitor of apoptosis proteins.
